# Tailored Exercise during Hematopoietic Stem Cell Transplantation Hospitalization in Children with Cancer: A Prospective Cohort Study

**DOI:** 10.3390/cancers12103020

**Published:** 2020-10-17

**Authors:** Javier S. Morales, Marta González Vicent, Pedro L. Valenzuela, Adrián Castillo-García, Elena Santana-Sosa, Alvaro Lassaletta, Alejandro Santos-Lozano, Carmen Fiuza-Luces, Alejandro Lucia

**Affiliations:** 1 Faculty of Sport Sciences, Universidad Europea de Madrid, 28041 Madrid, Spain; javier@fissac.com (J.S.M.); elena.santana@universidadeuropea.es (E.S.-S.); alejandro.lucia@universidadeuropea.es (A.L.); 2 Pediatric Hematology and Oncology Department, Hospital Infantil Universitario Niño Jesús, 28041 Madrid, Spain; mgonzalezv.hnjs@salud.madrid.org (M.G.V.); alvaro.lassaletta@salud.madrid.org (A.L.); 3 Department of Systems Biology, University of Alcalá, 28041 Madrid, Spain; pedrol.valenzuela@edu.uah.es; 4 Fissac—Physiology, Health and Physical Activity, 28041 Madrid, Spain; adrian@fissac.com; 5 i+HeALTH, Department of Health Sciences, European University Miguel de Cervantes, 47012 Valladolid, Spain; asantos@uemc.es; 6 Physical Activity and Health Laboratory, Instituto de Investigación Sanitaria Hospital ‘12 de Octubre’ (‘imas12′), 28041 Madrid, Spain

**Keywords:** immune reconstitution, graft-versus-host disease, childhood cancer, infection, exercise is medicine

## Abstract

**Simple Summary:**

The use of hematopoietic stem cell transplantation (HSCT), particularly among children, has increased over the last decades. However, it is frequently associated with a high risk of morbidity and mortality, and HSCT-related adverse effects can potentially impair survivors’ health status. We aimed to assess the effects on major clinical outcomes of a supervised exercise intervention performed by children with cancer during hospitalization for HSCT. The main finding of this prospective study in a quite large cohort of pediatric HSCT recipients (*n* = 118, aged 4–18 years) is that a moderate intensity supervised exercise program (aerobic + resistance exercises) performed from the beginning of the conditioning phase for HSCT until the end of the neutropenic phase is safe and well tolerated and tends to decrease risk of infections after allogeneic HSCT, as compared with not performing the program.

**Abstract:**

We assessed the clinical effects of a supervised exercise (aerobic + resistance) intervention performed during inpatient hospitalization for pediatric hematopoietic stem cell transplantation (HSCT). Patients were placed in an exercise (*n* = 65 (47 and 18 with allogeneic (allo-) and autologous (auto-) HSCT, respectively)) or a control (*n* = 53 (39 and 14)) group. Exercise interventions were performed in isolated hospital patient rooms. Patients were followed-up from the beginning of the conditioning phase up to 6 years. We assessed survival, risk of graft-versus-host disease (GvHD) or graft failure (primary outcomes), and engraftment kinetics, supportive care, toxicity profile, and immune reconstitution for auto-HSCT and allo-HSCT. The exercise intervention was safe and did not affect the risk of mortality, acute/chronic GvHD, or graft failure (all *p* > 0.05). No between-group differences (*p* > 0.05) were found for the remainder of clinical endpoints, except for a reduced number of total and viral infections in the exercise group after allo-HSCT (unadjusted *p* = 0.005 for both total and viral infections, and adjusted *p* = 0.023 and 0.083, respectively). In conclusion, exercise performed during inpatient hospitalization for pediatric HSCT is safe and well tolerated during both auto and allo-HSCT and tends to decrease the risk of infection after allo-HSCT. These findings provide additional support to the notion that a multidisciplinary approach (i.e., including the work of exercise specialists) is suitable in the management of children undergoing HSCT. Further studies are needed to determine whether applying a different training stimulus (notably, higher exercise intensities) exerts positive effects on HSCT prognosis in these patients.

## 1. Introduction

Hematopoietic stem cell transplantation (HSCT) is a treatment modality for a number of malignant and non-malignant conditions, and its use—particularly among children—has increased over the last three decades [1]. Cancer survivors treated with HSCT are at greater risk of re-hospitalization and mortality than their peers not receiving this treatment [2]. In addition, allogeneic HSCT (allo-HSCT) is frequently associated with a severe condition—graft-versus-host disease (GvHD)—with mortality rates ranging from 15% to 40% in the acute form of this condition (aGvHD), and from 30% to 50% in its chronic form (cGvHD) [3]. Thus, developing therapeutic strategies to reduce the morbidity and mortality associated with HSCT should be a priority. Given its multisystemic benefits, one potential strategy for managing the many chronic conditions associated with HSCT is physical exercise.

There is meta-analytic evidence that exercise improves functional capacity in children and adolescents treated for cancer in general [4], and undergoing HSCT in particular [5]. We previously reported preliminary evidence that supervised physical training might be beneficial for the immune system of children with cancer even when performed during the most aggressive phases of treatment [6] and HSCT-associated hospitalization [7,8,9]. We observed an attenuation in the reduction of dendritic cell count in children—most of them with leukemias—who performed physical exercise from the beginning of the HSCT conditioning phase until the end of the neutropenic phase, as compared with their non-exercised controls [9]. Whether exercise in pediatric HSCT recipients exerts beneficial effects on major clinical endpoints such as mortality, risk of GvHD or graft failure, or on HSCT-related adverse effects, is, however, unknown. Thus, the present study aimed to analyze the effects of supervised exercise during pediatric HSCT on survival and risk of a/cGvHD or of graft failure. Secondary outcomes were engraftment kinetics, supportive care (number of platelet/red blood cell transfusions, duration of fever, parenteral nutrition, and antibiotic treatment), toxicity profile, infections, and immune reconstitution at 15 and 30 days post-HSCT. We hypothesized that the exercise intervention would be safe and would improve overall health status by attenuating some of the HSCT-related adverse effects.

## 2. Results

### 2.1. Patient Characteristics

**Figure 1:**
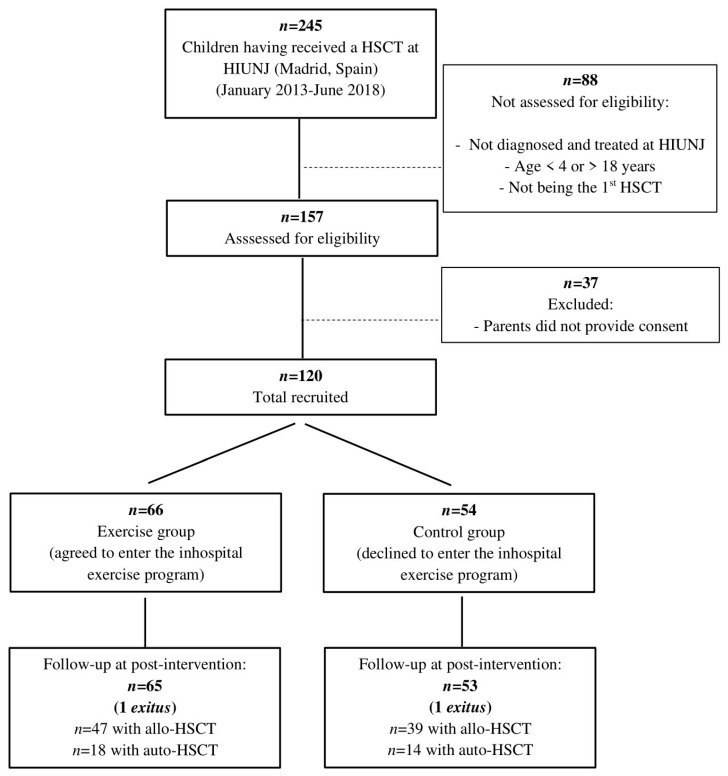
Flow diagram according to strengthening the reporting of observational studies in epidemiology statement. Abbreviations: allo-HSCT, allogeneic hematopoietic stem-cell transplantation; auto-HSCT, autologous hematopoietic stem-cell transplantation; HIUNJ, Hospital Infantil Universitario Niño Jesús; HSCT, hematopoietic stem cell transplantation.

A flow diagram of study participants is shown in Figure 1. A total of 120 children met all the eligibility criteria and entered the study. They were placed in a control (*n* = 54) or exercise (*n* = 66) group. Two participants (one per group) died during the neutropenic phase due to HSCT-related toxicities. Thus, the number of participants who completed the study from the beginning of the conditioning phase for HSCT until the end of the neutropenic phase was 53 (control (39 with allo-HSCT and 14 with autologous (auto-) HSCT)) and 65 (exercise group (47 and 18)). Baseline characteristics, including GvHD prophylaxis and the conditioning regimen preceding HSCT, did not differ between groups (Table 1). Similar results were obtained when comparisons between exercise and control groups were performed separately by type of HSCT, except for age at transplantation in allo-HSCT subgroups (Table S1).

The median follow-up from the start of the study was 2 (interquartile range (IQR) 3.8) years, with no between-group differences. Participants in the exercise group completed a median of 11 exercise sessions (IQR 13, minimum of 10 and maximum of 23 sessions), with individual variability in the number of sessions due to individual variations in the duration of the neutropenic phase (i.e., from a minimum of 10 days to a maximum of 30 days). No adverse effects or health problems attributable to the training sessions were recorded during the intervention.

### 2.2. Primary Outcomes

Kaplan-Meier analysis showed no between-group differences in the final cumulative survival probability for auto- (71% 95% confidence interval (41–88) vs. 67% (39–83) in the control and exercise group, respectively) (Figure S1A) or allo-HSCT (68% (50–80) vs. 74% (59–84)) (Figure S2A) or in cumulative probability for a second HSCT for auto- (free of HSCT: 93% (59–99) vs. 94% (67–92)) (Figure S1B) or allo-HSCT (75% (58–85) vs. 83% (69–91)) (Figure S2D).

No between-group differences were found for allo-HSCT in the final cumulative probability of aGvHD (free of aGvHD: 59% (42–72) in the control group vs. 51% (36–64) in the exercise group) (Figure S2B) or cGvHD (free of cGvHD: 89% (75–96) vs. 78% (64–88)) (Figure S2C). In fact, 41% and 10% of children in the control group and 48% and 21% of children in the exercise group developed aGvHD and cGvHD, respectively, with no between-group differences. No between-group differences were found for the remainder of primary outcomes for allo- or auto-HSCT (Table 2).

### 2.3. Secondary Outcomes

The effects of the exercise intervention on engraftment kinetics and supportive care are shown in Table 3 and Table 4, respectively. In unadjusted analyses, for allo-HSCT, we found a trend toward between-group differences in the number of days between HSCT and hospital discharge (*p* = 0.052) (Table 4). We found no between-group differences in toxicities (Table 5). The number of total and viral infections after allo-HSCT was significantly lower in the exercise than in the control group in unadjusted analysis (both *p* = 0.005) and tended to be lower after statistical adjustment (*p* = 0.023 and 0.083, respectively) (Table 6).

Analyses of immune reconstitution are shown in Table S2 (auto-HSCT) and Table S3 (allo-HSCT). We found no significant group by time interaction effect for any immune cell subtype.

## 3. Discussion

We assessed the effects of an in-hospital supervised exercise intervention performed during HSCT on survival, risk of GvHD or graft failure, engraftment kinetics, supportive care, toxicity profile, number of infections, and immune reconstitution in pediatric patients with cancer. The exercise intervention combined moderate-intensity aerobic and strength exercises (five weekly sessions of ~60 min) and lasted from the beginning of the conditioning phase for HSCT until the end of the neutropenic phase. Our main finding was that, although exercise had no overall effects—beneficial or harmful—on the majority of the analyzed parameters, it was safe and well tolerated. In addition, an interesting finding was the lower number of infections after allo-HSCT in the exercise group, which remained quasi-significant after statistical adjustment (*p* = 0.023 and 0.083 for total and viral infections, respectively). Further, in concordance with a recent study of our group in which fewer hospitalization days were observed for children and adolescents with cancer undergoing an exercise training program compared with a control group [11], we observed a trend (*p* = 0.052) in unadjusted analysis towards a lower number of days between allo-HSCT and hospital discharge in the exercise group (17 days) compared to the control group (21 days).

There is biological rationale to support that physical exercise interventions might help to lessen some of the side effects of pediatric HSCT. Previous findings support the feasibility of this type of intervention in children/adolescents undergoing HSCT, as it does not compromise the recovery of immune cells [9] and at the same time improves physical function [12,13]. Further, recent meta-analytic evidence indicates that physical exercise attenuates the functional decline of children and adolescents with cancer in general [4], and particularly in HSCT recipients [5], with functional decline being an often observed adverse effect in the context of pediatric cancer [14].

Beyond the effects on physical performance, physical exercise can also affect clinical outcomes. Infections are very common and serious adverse events in pediatric HSCT recipients [15]. We found that an exercise intervention tended to reduce infections after allo-HSCT, which is clinically important because infections––at least in the long term––are a leading cause of death, even in the absence of GvHD [16]. In this regard, a previous study found a lower mortality due to infections in adult allo-HSCT survivors who performed physical exercise during the peri-transplant period compared with their non-exercised peers [17]. The multisystemic benefits of regular exercise in general may also extend to the immune system, particularly the innate immune system [18]. Among the innate immune cell subtypes that can be potentially receptive to exercise, the evidence is especially strong for natural killer (NK) lymphocytes, which can show improved cytotoxicity (or ‘killing capacity’) [19]. Indeed, a moderate-intensity exercise intervention has been proven to increase NK cytotoxic activity in children undergoing HSCT, who are immune-compromised [7]. Notwithstanding, the evidence on the role of exercise on immune function in childhood cancer is inconclusive [20]. The biological mechanisms by which regular exercise might improve immune function, and particularly that of NK cells, remain elusive, although some candidate transcripts in peripheral blood mononuclear cells encoding ribosomal and oxidative phosphorylation proteins [21], or some transcriptomic changes (e.g., in translocation methylcytosine dioxygenase 1 (involved in DNA demethylation)), might be involved [22].

To achieve measurable training adaptations, exercise interventions should probably go beyond moderate intensity and include bouts of vigorous intensity, particularly for the fittest children [23]. However, the degree to which exercise intensity can influence the immune system of cancer patients is unclear. Based on the results of different studies, Nieman et al. proposed that regular moderate exercise lowers infection risk by enhancing immunosurveillance, whereas intensive physical exercise could lead to a reduction in immunosurveillance, and therefore to a potentially higher risk of infection [24,25,26]. Further evidence is needed—at least in cancer patients—to clarify whether intense exercise is really a stressor to immune function that could influence the risk of infections.

Several limitations must be noted in our study. First, there was heterogeneity in several participants’ characteristics (notably type of HSCT, graft manipulation method, and age). There was also variability in the number of exercise training sessions, largely due to the variability of the neutropenic phase. In this regard, the intervention was applied in a real-life scenario, where there are individual differences among patients. Another limitation is the fact that we did not perform a randomized controlled trial, which in any case would not have been feasible in our setting for ethical reasons. This could have biased patients’ enrollment, with the more active or fittest children more likely to enroll in the intervention group than their less fit or more inactive peers. In this regard, although we did not determine the participants’ physical activity levels before the study, we found no significant differences between groups for clinical characteristics, Karnofksy/Lanksy’s performance scores or body mass index—which is an indirect lifestyle indicator. In turn, there are main strengths in our study, including the relatively large sample size compared with previous exercise interventional research in the field, as well as the novelty of our approach. Moreover, to our knowledge, this is the first study that performs an in-depth assessment of clinical outcomes (including risk of GvHD) after an exercise intervention conducted from the beginning of the conditioning regimen until the end of the neutropenic phase in pediatric HSCT.

## 4. Materials and Methods

### 4.1. Participants

This study followed a concurrent prospective cohort design and was performed in the Hospital Infantil Universitario Niño Jesús (HIUNJ, Madrid, Spain) in adherence to the Declaration of Helsinki. It was approved by the local Ethics Committee (approval number R-0007/13) and was performed following the Strengthening the Reporting of Observational Studies in Epidemiology statement. All participants and their parents or legal guardians gave their written informed consent to participate in the study (which took place from January 2013 to June 2019. We used the following inclusion criteria: (i) aged 4–18 years (both sexes); (ii) diagnosed, treated and followed at the aforementioned hospital; and (iii) being in an isolated unit during the neutropenic phase—with high-efficiency particulate air filter and room positive pressure. Because we used a convenience sample, no sample size calculation was done a priori.

Since 2013, the HIUNJ offers all patients aged ≥4 years who are under treatment in the pediatric oncology-hematology unit to enter a supervised exercise program to be performed inside this center—pending approval by the oncologist/s in charge. Participants were placed in an exercise or control group attending to whether they and their parents or legal guardians had freely decided to participate or not in the program during HSCT. A follow-up of up to 6 years was used to analyze the risk of mortality, a/cGvHD, or graft failure.

### 4.2. Supervised Exercise Intervention

The exercise intervention (duration ~3 weeks) started at the beginning of the conditioning regimen and lasted until neutrophil engraftment (i.e., the end of the neutropenic phase (where an absolute neutrophil count >0.5 × 10^9^/L must be reached)). We used a combined (aerobic and resistance exercises) training design. Participants performed the training sessions inside their own isolated room. All sessions were individually supervised by a graduate fitness specialist with a strong background in pediatric exercise. All the training equipment was sterilized before each session performed during the neutropenic phase, with fitness instructors wearing facemasks.

The program included five weekly sessions of ~60-min duration. Each session started with a 10-min warm-up (cycle ergometer exercise at very low intensities and stretching of the major muscle groups) and ended with a cool-down of the same characteristics. The aerobic phase (~25 min duration) consisted of cycle-ergometer (Rhyno Magnetic H490; BH Fitness Proaction, Vitoria, Spain) (Video S1) or arm cranking exercise—in those children with an amputee lower limb (Monark Rehab Trainer model 881E; Monark, Varberg, Sweden). The training load was gradually increased depending on the patients’ age, physical capacity, and health status. Exercise intensity was recorded continuously with heart rate (HR) monitors (Xtrainer Plus; Polar Electro OY, Kempele, Finland) and progressively increased from 65% to 80% of HR reserve (i.e., age-predicted maximum HR (220 minus age, in years) minus supine resting HR) [11]. Thereafter, participants performed strength exercises engaging major muscle groups (leg extension, half squat, plank on knees, supine bridge, arm curl, elbow extension, push-ups, and rowing) for a total duration of ~15 min (Videos S2 and S3). They performed three sets of 12–15 repetitions per exercise, with 1-min rest between sets, using their own body weight (e.g., for planks), elastic bands (usually for the youngest children), or barbells (for the oldest ones). The load (i.e., resistance of elastic bands and weight of barbells) was gradually increased as the participants became stronger during the program. A session was deemed complete when at least 90% of the prescribed exercises were done successfully [27].

Patients were clinically assessed before every training session. Thus, any session was cancelled when the clinician in charge decided that the poor health status of the patient contraindicated acute exercise (e.g., if a child had platelet or hemoglobin levels <10,000/μL or <8 g/dL, respectively, temperature ≥38 °C, severe muscle pain, diarrhea, hemorrhage, or extreme fatigue).

### 4.3. Outcomes

#### 4.3.1. Primary Outcomes

We collected data on mortality, development of a/cGvHD, or new HSCT from medical records.

#### 4.3.2. Secondary Outcomes

We also recorded from medical records data on engraftment kinetics (days to neutrophil engraftment and to platelet counts ≥20, ≥50, and ≥100 × 10^9^/L, respectively, and days of myelosuppression post-HSCT), supportive care (number of platelet/red blood cell transfusions, duration of fever, parenteral nutrition and antibiotic treatment), toxicity profile (mucositis, vomiting, diarrhea, engraftment syndrome, hemorrhagic cystitis, neurologic, liver and renal toxicity), and number and type (viral, bacterial or fungal) of infections per child after HSCT. We assessed immune reconstitution (leukocyte, neutrophil, monocyte, lymphocyte, and lymphocyte populations (T-lymphocytes, CD4+ and CD4 subsets, CD8+ and CD8 subsets, NK and NK subtypes (NK^dim^ and NK^bright^)) and dendritic cells) at the beginning of the conditioning phase and on days 15 and 30 post-HSCT on fresh whole blood samples using multiparametric flow cytometry (FACS Canto II; Becton Dickinson, Madrid, Spain).

### 4.4. Statistical Analysis

We performed separate analyses attending to type of HSCT (allo-HSCT or auto-HSCT). We assessed between-group differences at baseline using unpaired Student’s *t* tests or χ^2^ tests for continuous or dichotomous variables, respectively, and between-group differences in continuous endpoint measures by comparing the intra-individual score differences from baseline to hospital discharge in the two groups (control and exercise). We used analysis of covariance (ANCOVA) to compare the mean differences in continuous endpoint measures between the two groups [28]. We used binary logistic regression to compare the risk of a/cGvHD (for allo-HSCT), graft failure, death, toxicity, and infections after HSCT for allo- and auto-HSCT. Linear mixed models for repeated-measures were used to assess group by time interaction in immune reconstitution. To minimize the risk of type I error, we corrected all the analyses for multiple comparisons with the stringent Bonferroni method (i.e., dividing 0.05 by the number of comparisons). We performed Kaplan-Meier analysis to assess between-group differences in the distribution of survival, second HSCT (for allo- and auto-HSCT), and incidence of a/cGvHD (for allo-HSCT).

We adjusted the analyses of allo-HSCT by group (i.e., exercise or control), graft manipulation (i.e., manipulated or unmanipulated), age at HSCT, sex differences between donor and recipient (i.e., yes or not), conditioning regimen (i.e., myeloablative or nonmyeloablative), source (i.e., peripheral blood or umbilical cord) and origin (i.e., parent, sibling or unrelated donor) of donor cells, GvHD prophylaxis (i.e., cyclosporine or cyclosporine + methylprednisolone), disease status (i.e., 1st, 2nd, >2nd complete remission or not in remission), and human leukocyte antigen (HLA) match status (i.e., HLA-matched and related, HLA-matched and unrelated or HLA-mismatched related or unrelated). With the exception of GvHD prophylaxis, HLA match status, sex differences between donor and recipient, graft manipulation and donor of cells, we used all the aforementioned covariates for adjusting the analyses of auto-HSCT. All statistical analyses were conducted using SPSS version 23.0 (SPSS Inc., Chicago, IL, USA).

## 5. Conclusions

A moderate-intensity supervised exercise intervention performed during inpatient hospitalization appears to be safe for children and adolescents undergoing HSCT. Importantly, a tendency towards a lower number of total infections after allo-HSCT was observed with the exercise intervention. Further research on physical exercise and childhood cancer, and particularly HSCT, is needed to define whether applying a different training stimulus (e.g., increasing intensity of exercise training) might exert positive effects on auto- and allo-HSCT prognosis in these patients.

## Figures and Tables

**Table 1:** Main demographic and clinical characteristics by group.

Variable	Control Group (*n* = 53)	Exercise Group (*n* = 65)	*p*-Value
Age (mean ± SD [range], years)			
At diagnosis	9 ± 4 (4, 17)	10 ± 4 (4, 17)	0.070
At HSCT	10 ± 4 (4, 18)	11 ± 4 (5, 18)	0.081
Sex (% male)			
Recipient	64%	63%	0.904
Donor	50%	46%	0.751
Diagnosis (%)			
ALL/other leukemias	70/30%	68/32%	0.805
Disease status (%)			
1st CR	28%	32%	0.638
2nd CR	36%	34%	0.820
>2nd CR	17%	22%	0.534
Not in remission	19%	12%	0.324
Type of HSCT (%)			
Allogeneic/autologous	74/26%	72/28%	0.877
Source of donor cells (%)			
Peripheral blood/umbilical cord	98/2%	94/6%	0.377
Origin of cells in allo-HSCT (%)			
Parent	62%	60%	0.853
Sibling	15%	23%	0.353
Unrelated donor	23%	17%	0.483
Conditioning regimen (%)			
Myeloablative/Nonmyeloablative	91/9%	88/12%	0.599
GvHD prophylaxis in allo-HSCT (%)			
Cyclosporine	28%	23%	0.650
Cyclosporine + Methylprednisolone	72%	77%	0.650
HLA-match status in allo-HSCT (%)			
HLA-matched and related	8%	15%	0.300
HLA-matched and unrelated	18%	11%	0.330
HLA-mismatched (related or unrelated)	74%	74%	0.991
Graft manipulation method (%)			
Manipulated/Unmanipulated	73/27%	74/26%	0.778
HCT-CI * (median [range])	0 (0, 1)	0 (0, 2)	0.197
Anthropometrical variables (mean ± SD)			
Body weight (kg)	37.4 ± 22.8	39.6 ± 15	0.541
BMI (kg/m^2^)	19.1 ± 5.3	18.5 ± 3.5	0.459
Karnofsky/Lansky’s performance scale ** (mean ± SD)	93 ± 6	93 ± 8	0.726

Abbreviations: ALL, acute lymphoblastic leukemia; allo-HSCT, allogeneic hematopoietic stem-cell transplantation; BMI, body mass index; CR, complete remission; GvHD, graft-versus-host disease; HCT-CI, hematopoietic cell transplantation-comorbidity index; HLA, human leukocyte antigens; HSCT, hematopoietic stem cell transplantation; SD, standard deviation. Symbol: * comorbidities were calculated using the HCT-CI [10]; ** the Karnofsky and Lansky scales for Pediatric Functional Status were used to assess the performance status of participants aged ≥16 or <16 years, respectively, on a 0 to 100 (“perfect”) scale.

**Table 2:** Effects of exercise intervention on primary outcomes.

**Allogeneic-HSCT**						
	**Control Group** **(*n* = 39)**		**Exercise Group** **(*n* = 47)**			
**Outcome**	**Unadjusted**	Adjusted *	**Unadjusted**	Adjusted *	**Unadjusted between-group Difference ** ***p*-value**	Adjusted * between-group Difference ***p*-value**
Mortality						
OR (95% CI) for death	1.00 (Reference)	1.00 (Reference)	0.78 (0.46, 2.77)	2.55 (0.65, 9.98)	0.784	0.179
Time since HSCT to death (days)	276 ± 111 (50, 503)	396 ± 373 (364, 1256)	393 ± 97 (195, 591)	351 ± 258 (243, 947)	0.436	0.915
HSCT						
Number of HSCT	1 ± 0 (1, 1)	1 ± 0 (1, 2)	1 ± 0 (1, 1)	1 ± 0 (1, 2)	0.293	0.556
Graft failure (OR (95% CI))	1.00 (Reference)	1.00 (Reference)	0.60 (0.21, 1.69)	0.57 (0.13, 2.49)	0.331	0.459
Time from first to second HSCT (days)	256 ± 67 (114, 398)	358 ± 85 (226, 1441)	298 ± 75 (139, 456)	455 ± 51 (189, 1099)	0.684	0.587
GvHD						
Risk of aGvHD, (OR (95% CI))	1.00 (Reference)	1.00 (Reference)	1.38 (0.59, 3.25)	1.59 (0.49, 5.20)	0.464	0.441
Grade of aGvHD	2 ± 0 (2, 3)	1 ± 1 (−1, 3)	2 ± 0 (2, 3)	1 ± 1 (−1, 3)	0.464	0.521
Time from HSCT to aGvHD, (days)	40 ± 6 (29, 52)	58 ± 19 (18, 98)	39 ± 5 (30, 49)	59 ± 22 (11, 106)	0.876	0.953
Risk of cGvHD (OR (95% CI))	1.00 (Reference)	1.00 (Reference)	2.37 (0.68, 8.24)	2.16 (0.51, 9.22)	0.177	0.298
Grade of cGvHD	2 ± 0 (1, 2)	1 ± 0 (1, 2)	1 ± 0 (1, 2)	1 ± 0 (0, 2)	0.271	0.542
Time from HSCT to cGvHD (days)	116 ± 39 (31, 201)	72 ± 93 (63, 307)	137 ± 25 (83, 191)	103 ± 61 (91, 297)	0.665	0.220
**Autologous-HSCT**						
	**Control Group** **(*n* = 14)**		**Exercise Group** **(*n* = 18)**			
	**Unadjusted**	Adjusted **	**Unadjusted**	Adjusted **	**Unadjusted between-group Difference ** ***p*-value**	Adjusted ** between-group Difference ***p*-value**
Mortality						
OR (95% CI) for death	1.00 (Reference)	1.00 (Reference)	0.75 (0.09, 6.11)	0.60 (0.61, 5,88)	0.788	0.658
Time since HSCT to death (days)	1232 ± 514 (981, 3445)	*N*/A	528 ± 514 (486, 2741)	*N*/A	0.435	*N*/A
HSCT						
Number of HSCT	1 ± 0 (1, 1)	1 ± 0 (1, 1)	1 ± 0 (1, 1)	1 ± 0 (1, 1)	0.860	0.952
Graft failure (OR (95% CI))	1.00 (Reference)	1.00 (Reference)	0.77 (0.04, 13.41)	1.4 (0.04, 49.88)	0.854	0.854
Time from first to second HSCT (days)	*N*/A	*N*/A	*N*/A	*N*/A	*N*/A	N/A

All data are expressed as mean ± SEM (95% confidence interval) unless otherwise stated. The control group was used as reference for regression analyses. We corrected all the analyses for multiple comparisons with the Bonferroni method; that is, dividing 0.05 by the number of comparisons. Thus, the threshold *p*-value for statistical significance was set at 0.0045 (=0.05/11) for allogeneic-HSCT and 0.01 (=0.05/5) for autologous-HSCT. Abbreviations: aGvHD, acute GvHD; cGvHD, chronic GvHD; CI, confidence interval; HSCT, hematopoietic stem cell transplantation; GvHD, graft-versus-host-disease; N/A, not available; OR, odds ratio. Symbols: * adjusted by group (i.e., exercise or control), graft manipulation (i.e., manipulated or unmanipulated), age at HSCT, sex differences between donor and recipient (i.e., yes or no), conditioning regimen (i.e., myeloablative or nonmyeloablative), source (i.e., peripheral blood or umbilical cord) and origin (i.e., parent, sibling or unrelated donor) of donor cells, GvHD prophylaxis (i.e., cyclosporine or cyclosporine + methylprednisolone), disease status (i.e., 1st, 2nd, >2nd complete remission or not in remission) and human leukocyte antigens match status (i.e., HLA-matched and related, HLA-matched and unrelated, or HLA-mismatched (related or unrelated)); ** adjusted by group, age at HSCT, conditioning regimen, source of donor cells and disease status (those mentioned above for allo-HSCT).

**Table 3:** Effects of exercise intervention on secondary outcomes (engraftment kinetics).

**Allogeneic-HSCT**						
	**Control Group** **(*n* = 39)**		**Exercise Group** **(*n* = 47)**			
**Outcome**	**Unadjusted**	Adjusted *	**Unadjusted**	Adjusted *	**Unadjusted between-group Difference** ***p*-** **Value**	Adjusted * between-group Difference ***p*-Value**
Time to neutrophil engraftment (days)	16 ± 1 (14, 17)	15 ± 2 (11, 19)	14 ± 1 (13, 16)	13 ± 2 (9, 17)	0.126	0.170
Time to platelet count ≥ 20 × 10^9^/L (days)	13 ± 1 (11, 15)	13 ± 3 (7, 19)	12 ± 1 (10, 14)	11 ± 3 (6, 17)	0.368	0.454
Time to platelet count ≥ 50 × 10^9^/L (days)	15 ± 1 (12, 18)	21 ± 4 (13, 29)	14 ± 1 (12, 17)	19 ± 4 (11, 27)	0.857	0.489
Time to platelet count ≥ 100 × 10^9^/L (days)	19 ± 3 (14, 25)	73 ± 8 (56, 89)	22 ± 2 (17, 27)	73 ± 7 (58, 88)	0.382	0.960
Myelosuppression (days)	12 ± 1 (10, 14)	11 ± 3 (4, 17)	10 ± 1 (8, 12)	9 ± 3 (3, 16)	0.329	0.563
**Autologous-HSCT**						
	**Control Group** **(*n* = 14)**		**Exercise Group** **(*n* = 18)**			
	**Unadjusted**	Adjusted **	**Unadjusted**	Adjusted **	**Unadjusted between-Group Difference** ***p*-Value**	Adjusted ** between-Group Difference ***p*-Value**
Time to neutrophil engraftment (days)	13 ± 1 (12, 14)	13 ± 1 (12, 14)	12 ± 0 (11, 13)	13 ± 1 (12, 14)	0.403	0.920
Time to platelet count ≥ 20 × 10^9^/L (days)	10 ± 1 (8, 13)	10 ± 2 (7, 14)	12 ± 1 (11, 13)	13 ± 1 (10, 16)	0.251	0.282
Time to platelet count ≥ 50 × 10^9^/L (days)	13 ± 1 (10, 17)	12 ± 1 (7, 18)	15 ± 1 (12, 17)	16 ± 1 (11, 20)	0.460	0.195
Time to platelet count ≥ 100 × 10^9^/L (days)	16 ± 4 (15, 19)	*N*/A	19 ± 3 (16, 21)	*N*/A	0.667	*N*/A
Myelosuppression (days)	7 ± 1 (6, 8)	7 ± 1 (5, 8)	8 ± 1 (7, 9)	9 ± 1 (7, 10)	0.135	0.071

All data are expressed as mean ± SEM (95% confidence interval). We corrected all the analyses for multiple comparisons with the Bonferroni method; that is, dividing 0.05 by the number of comparisons. Thus, the threshold *p*-value for statistical significance was set at 0.01 (=0.05/5) for both allogeneic and autologous HSCT. Abbreviations: HSCT, hematopoietic stem cell transplantation; *N*/A, not available. Symbol: * adjusted by group (i.e., exercise or control), graft manipulation (i.e., manipulated or unmanipulated), age at hematopoietic stem cell transplantation, sex differences between donor and recipient (i.e., yes or no), conditioning regimen (i.e., myeloablative or nonmyeloablative), source (i.e., peripheral blood or umbilical cord) and origin (i.e., parent, sibling or unrelated donor) of donor cells, graft-versus-host-disease prophylaxis (i.e., cyclosporine or cyclosporine + methylprednisolone), disease status (i.e., 1st, 2nd, >2nd complete remission or not in remission) and human leukocyte antigens match status (i.e., HLA-matched and related, HLA-matched and unrelated, or HLA-mismatched (related or unrelated)); ** adjusted by group, age at HSCT, conditioning regimen, source of donor cells and disease status (those mentioned above for allo-HSCT).

**Table 4:** Effects of exercise intervention on secondary outcomes (supportive care).

**Allogeneic-HSCT**						
	**Control Group** **(*n* = 39)**		**Exercise Group** **(*n* = 47)**			
**Outcome**	**Unadjusted**	Adjusted *	**Unadjusted**	Adjusted *	**Unadjusted between-group Difference *p*-value**	Adjusted * between-group Difference *p*-value
Platelet transfusions (number)	3 ± 1 (2, 5)	3 ± 2 (1, 6)	3 ± 1 (2, 4)	3 ± 2 (0, 6)	0.758	0.474
Red blood cell transfusions (number)	3 ± 0 (2, 4)	2 ± 1 (1, 4)	2 ± 0 (1, 3)	2 ± 1 (1, 4)	0.147	0.546
Fever (days)	3 ± 0 (2, 4)	1 ± 1 (0, 3)	2 ± 0 (1, 3)	0 ± 1 (0, 2)	0.108	0.040
Antibiotic treatment (days)	18 ± 2 (15, 22)	18 ± 4 (9, 26)	16 ± 2 (13, 19)	14 ± 4 (6, 22)	0.256	0.206
Parenteral nutrition (days)	6 ± 2 (2, 10)	7 ± 4 (1, 15)	6 ± 2 (3, 10)	7 ± 4 (1, 15)	0.862	0.787
Time between HSCT and hospital discharge (days)	21 ± 1 (18, 24)	20 ± 4 (12, 28)	17 ± 1 (14, 20)	15 ± 4 (8, 23)	0.052	0.082
**Autologous-HSCT**						
	**Control Group** **(*n* = 14)**		**Exercise Group** **(*n* = 18)**			
	**Unadjusted**	Adjusted **	**Unadjusted**	Adjusted **	**Unadjusted between-group Difference** ***p*-value**	Adjusted ** between-group Difference ***p*-value**
Platelet transfusions (number)	4 ± 1 (3, 5)	4 ± 1 (3, 5)	3 ± 0 (2, 4)	3 ± 1 (2, 5)	0.099	0.566
Red blood cell transfusions (number)	2 ± 0 (2, 2)	2 ± 0 (1, 3)	2 ± 0 (1, 2)	2 ± 0 (1, 2)	0.141	0.371
Fever (days)	1 ± 0 (1, 2)	1 ± 0 (1, 2)	1 ± 0 (1, 2)	1 ± 0 (0, 2)	0.690	0.311
Antibiotic treatment (days)	12 ± 1 (10, 14)	12 ± 1 (10, 14)	13 ± 1 (11, 15)	14 ± 1 (12, 16)	0.703	0.242
Parenteral nutrition (days)	10 ± 2 (6, 13)	9 ± 1 (6, 11)	9 ± 2 (5, 12)	8 ± 1 (6, 11)	0.595	0.793
Time between HSCT and hospital discharge (days)	15 ± 1 (14, 17)	16 ± 1 (14, 17)	16 ± 1 (15, 18)	17 ± 1 (16, 18)	0.243	0.158

All data are expressed as mean ± SEM (95% confidence interval). We corrected all the analyses for multiple comparisons with the Bonferroni method; that is, dividing 0.05 by the number of comparisons. Thus, the threshold *p*-value for statistical significance was set at 0.008 (=0.05/6) for both allogeneic and autologous HSCT. Abbreviation: HSCT, hematopoietic stem cell transplantation. Symbols: * adjusted by group (i.e., exercise or control), graft manipulation (i.e., manipulated or unmanipulated), age at HSCT, sex differences between donor and recipient (i.e., yes or no), conditioning regimen (i.e., myeloablative or nonmyeloablative), source (i.e., peripheral blood or umbilical cord) and origin (i.e., parent, sibling or unrelated donor) of donor cells, graft-versus-host-disease prophylaxis (i.e., cyclosporine or cyclosporine + methylprednisolone), disease status (i.e., 1st, 2nd, >2nd complete remission or not in remission) and human leukocyte antigens match status (i.e., HLA-matched and related, HLA-matched and unrelated, or HLA-mismatched (related or unrelated)); ** adjusted by group, age at HSCT, conditioning regimen, source of donor cells and disease status (those mentioned above for allo-HSCT).

**Table 5:** Effects of exercise intervention on secondary outcomes (toxicity profile, any grade).

**Allogeneic-HSCT**						
	**Control Group** **(*n* = 39)**		**Exercise Group** **(*n* = 47)**			
**Risk of Outcomes**	**Unadjusted**	Adjusted *	**Unadjusted**	Adjusted *	**Unadjusted between-group Difference** ***p*-Value**	Adjusted * between-group Difference ***p*-Value**
Mucositis	1.00 (Reference)	1.00 (Reference)	0.89 (0.35, 2.23)	1.57 (0.45, 5.50)	0.802	0.478
Vomiting	1.00 (Reference)	1.00 (Reference)	0.84 (0.34, 2.07)	0.80 (0.23, 2.78)	0.705	0.729
Diarrhea	1.00 (Reference)	1.00 (Reference)	1.45 (0.58, 3.66)	1.21 (0.34, 4.26)	0.430	0.764
Engraftment syndrome	1.00 (Reference)	1.00 (Reference)	0.79 (0.33, 1.92)	2.53 (0.66, 9.70)	0.607	0.176
Hemorrhagic cystitis	1.00 (Reference)	1.00 (Reference)	0.50 (0.13, 1.94)	0.74 (0.12, 4.46)	0.312	0.741
Neurologic toxicity	1.00 (Reference)	1.00 (Reference)	0.89 (0.38, 2.13)	1.02 (0.24, 4.34)	0.797	0.982
Liver toxicity	1.00 (Reference)	1.00 (Reference)	0.39 (0.13, 1.14)	0.29 (0.06, 1.39)	0.086	0.121
Renal toxicity	1.00 (Reference)	1.00 (Reference)	0.97 (0.34, 2.74)	0.83 (0.21, 3.33)	0.953	0.789
**Autologous-HSCT**						
	**Control Group** **(*n* = 14)**		**Exercise Group** **(*n* = 18)**			
	**Unadjusted**	Adjusted **	**Unadjusted**	Adjusted **	**Unadjusted between-Group Difference** ***p*-Value**	Adjusted ** between-group Difference ***p*-Value**
Mucositis	1.00 (Reference)	1.00 (Reference)	1.36 (0.23, 8.08)	2.23 (0.14, 34.98)	0.733	0.569
Vomiting	1.00 (Reference)	1.00 (Reference)	4.50 (0.72, 28.15)	2.83 (0.35, 22.56)	0.108	0.327
Diarrhea	1.00 (Reference)	1.00 (Reference)	2.24 (0.45, 11.11)	1.96 (0.28, 13.64)	0.324	0.498
Engraftment syndrome	1.00 (Reference)	1.00 (Reference)	2.19 (0.47, 10.21)	2.76 (0.39, 19.34)	0.319	0.307
Hemorrhagic cystitis	1.00 (Reference)	1.00 (Reference)	2.38 (0.42, 13.39)	7.16 (0.59, 87.28)	0.325	0.123
Neurologic toxicity	1.00 (Reference)	1.00 (Reference)	1.50 (0.17, 12.94)	2.65 (0.15, 48.29)	0.712	0.511
Liver toxicity	1.00 (Reference)	1.00 (Reference)	4.40 (0.77, 25.15)	10.84 (0.70, 167.13)	0.096	0.088
Renal toxicity	1.00 (Reference)	1.00 (Reference)	2.22 (0.37, 13.54)	3.84 (0.43, 34.13)	0.386	0.228

All data are expressed as odds ratio (95% confidence interval) in the exercise group with the control group used as reference for regression analyses. We corrected all the analyses for multiple comparisons using the stringent Bonferroni method; that is, dividing 0.05 by the number of comparisons. Thus, the threshold *p*-value for statistical significance was set at 0.006 (=0.05/8) for both allogeneic and autologous HSCT. Abbreviation: HSCT, hematopoietic stem cell transplantation. Symbols: * adjusted for group (i.e., exercise or control), graft manipulation (i.e., manipulated or unmanipulated), age at HSCT, sex differences between donor and recipient (i.e., yes or no), conditioning regimen (i.e., myeloablative or nonmyeloablative), source (i.e., peripheral blood or umbilical cord) and origin (i.e., parent, sibling or unrelated donor) of donor cells, graft-versus-host-disease prophylaxis (i.e., cyclosporine or cyclosporine + methylprednisolone), disease status (i.e., 1st, 2nd, >2nd complete remission or not in remission) and human leukocyte antigens match status (i.e., HLA-matched and related, HLA-matched and unrelated, or HLA-mismatched (related or unrelated)); ** adjusted for group, age at HSCT, conditioning regimen, source of donor cells and disease status (those mentioned above for allo-HSCT).

**Table 6:** Effects of exercise intervention on secondary outcomes (number of infections per child).

**Allogeneic-HSCT**						
	**Control Group** **(*n* = 39)**		**Exercise Group** **(*n* = 47)**			
**Outcome**	**Unadjusted**	Adjusted *	**Unadjusted**	Adjusted *	**Unadjusted between-group Difference** ***p*-Value**	Adjusted * between-group Difference ***p*-Value**
Number of infections after HSCT	2 ± 0 (1, 2)	2 ± 0 (1, 3)	1 ± 0 (1, 1)	2 ± 0 (1, 2)	0.005	0.023
Number of viral infections after HSCT	1 ± 0 (1, 1)	1 ± 0 (0, 2)	0 ± 0 (0, 1)	1 ± 0 (0, 1)	0.005	0.083
Number of bacterial infections after HSCT	0 ± 0 (0, 1)	1 ± 0 (0, 1)	0 ± 0 (0, 1)	1 ± 0 (0, 1)	0.947	0.942
Number of fungal infections after HSCT	1 ± 0 (0, 1)	1 ± 0 (0,1)	0 ± 0 (0, 0)	0 ± 0 (0, 1)	0.022	0.026
**Autologous-HSCT**						
	**Control Group** **(*n* = 14)**		**Exercise Group** **(*n* = 18)**			
	**Unadjusted**	Adjusted **	**Unadjusted**	Adjusted **	**Unadjusted between-group Difference** ***p*-value**	Adjusted ** between-group Difference ***p*-value**
Number of infections after HSCT	1 ± 0 (1, 2)	1 ± 0 (1, 2)	1 ± 0 (0, 1)	1 ± 0 (0, 1)	0.273	0.213
Number of viral infections after HSCT	1 ± 0 (0, 1)	0 ± 0 (0, 1)	0 ± 0 (0, 1)	0 ± 0 (0, 1)	0.678	0.959
Number of bacterial infections after HSCT	0 ± 0 (0, 1)	1 ± 0 (0, 1)	0 ± 0 (0, 1)	0 ± 0 (0, 1)	0.525	0.230
Number of fungal infections after HSCT	0 ± 0 (0, 0)	0 ± 0 (0, 0)	0 ± 0 (0, 0)	0 ± 0 (0, 0)	0.518	0.728

All data are expressed as mean ± SEM (95% confidence interval). The control group was used as reference for regression analyses. We corrected all the analyses for multiple comparisons with the Bonferroni method; that is, dividing 0.05 by the number of comparisons. Thus, the threshold *p*-value for statistical significance was set at 0.0125 (=0.05/4) for both allogeneic and autologous HSCT and significant differences between groups are in bold. Abbreviations: CI, confidence interval; HSCT, hematopoietic stem cell transplantation; Symbols: * adjusted by group (i.e., exercise or control), graft manipulation (i.e., manipulated or unmanipulated), age at HSCT, sex differences between donor and recipient (i.e., yes or no), conditioning regimen (i.e., myeloablative or nonmyeloablative), source (i.e., peripheral blood or umbilical cord) and origin (i.e., parent, sibling or unrelated donor) of donor cells, GvHD prophylaxis (i.e., cyclosporine or cyclosporine + methylprednisolone), disease status (i.e., 1st, 2nd, >2nd CR or not in remission) and human leukocyte antigens match status (i.e., HLA-matched and related, HLA-matched and unrelated, or HLA-mismatched (related or unrelated)); ** adjusted by group, age at HSCT, conditioning regimen, source of donor cells and disease status (those mentioned above for allo-HSCT).

## Supplementary Materials

The following are available online at https://www.mdpi.com/2072-6694/12/10/3020/s1. Video S1: Cycle-ergometer training in a study participant; Video S2: Example of strength exercise (arm curl) in a study participant; Video S3: Example of strength exercise (push-up) in a study participant; Table S1: Main demographic and clinical characteristics of study patients by group and type of HSCT; Table S2: Analysis of immune reconstitution in patients undergoing autologous hematopoietic stem cell transplantation; Table S3: Analysis of immune reconstitution in patients undergoing allogeneic hematopoietic stem cell transplantation; Figure S1: Kaplan-Meier analysis of cumulative probability of survival after autologous hematopoietic stem cell transplantation (auto-HSCT) (A) and undergoing a second auto-HSCT (B). Figure S2: Kaplan-Meier analysis of cumulative probability of survival after allogeneic hematopoietic stem cell transplantation (allo-HSCT) (A), having acute (B) or chronic graft-versus-host-disease (C), or undergoing a second allo-HSCT (D).
