# Supplementary material for: Tailored Exercise during Hematopoietic Stem Cell Transplantation Hospitalization in Children with Cancer: A Prospective Cohort Study
Source: Cancers (Basel). 2020 Oct 17;12(10):3020. doi: 10.3390/cancers12103020 (PMC7650695; doi:10.3390/cancers12103020)
Supplement: Supplementary file 1 [file cancers-12-03020-s001.zip › R1 - Table S1.docx]

**Table S1**. Main demographic and clinical characteristics of study patients by group and type of transplant.

|  | **Allo-HSCT** | | |  | **Auto-HSCT** | | |
| --- | --- | --- | --- | --- | --- | --- | --- |
| **Variable** | **Control**  **(n=39)** | **Exercise**  **(n=47)** | ***P*-value between groups** |  | **Control**  **(n=14)** | **Exercise**  **(n=18)** | ***P*-value between groups** |
| **Age (mean ± SD [range], years)** |  |  |  |  |  |  |  |
| At diagnosis | 9 ± 4 (4, 17) | 10 ± 4 (4, 17) | 0.133 |  | 10 ± 4 (4, 17) | 10 ± 4 (4, 16) | 0.938 |
| At HSCT | 10 ± 4 (5, 17) | 11 ± 4 (5, 18) | **0.025** |  | 11 ± 5 (5, 18) | 11 ± 4 (5, 17) | 0.820 |
| **Sex (% male)** |  |  |  |  |  |  |  |
| Recipient | 64% | 64% | 1.000 |  | 64% | 61% | 1.000 |
| Donor | 50% | 46% | 0.811 |  | - | - | N/A |
| **Diagnosis (%)** |  |  |  |  |  |  |  |
| ALL/other leukemias | 67/33% | 64/36% | 0.823 |  | 21/79% | 33/67% | 0.694 |
| **Disease status (%)** |  |  |  |  |  |  |  |
|  |  |  |  |  |  |  |  |
| 1^st^ CR | 28% | 28% | 0.955 |  | 13% | 25% | 0.471 |
| 2^nd^ CR | 36% | 32% | 0.697 |  | 16% | 22% | 1.000 |
| >2^nd^ CR | 18% | 25% | 0.399 |  | 6% | 6% | 1.000 |
| Not in remission | 18% | 15% | 0.702 |  | 9% | 3% | 0.295 |
| **Source of donor cells (%)** |  |  |  |  |  |  |  |
| Peripheral blood/umbilical cord | 97/3% | 96/4% | 1.000 |  | 100/0% | 89/11% | 0.492 |
| **Origin of cells (%)** |  |  |  |  |  |  |  |
| Parent | 62% | 60% | 0.853 |  | - | - | N/A |
| Sibling | 15% | 23% | 0.353 |  | - | - | N/A |
| Unrelated donor | 23% | 17% | 0.483 |  | - | - | N/A |
| **Conditioning regimen (%)** |  |  |  |  |  |  |  |
| Myeloablative/Nonmyeloablative | 94/6% | 95/5% | 0.847 |  | 71/29% | 75/25% | 0.825 |
| **GvHD prophylaxis (%)** |  |  |  |  |  |  |  |
| Cyclosporine | 28% | 23% | 0.650 |  | - | - | N/A |
| Cyclosporine + Methylprednisolone | 72% | 77% | 0.650 |  | - | - | N/A |
| **HLA match status (%)** |  |  |  |  |  |  |  |
| HLA-matched and related | 8% | 15% | 0.300 |  | - | - | N/A |
| HLA-matched and unrelated | 18% | 11% | 0.330 |  | - | - | N/A |
| HLA-mismatched (related or unrelated) | 74% | 74% | 0.991 |  | - | - | N/A |
| **Graft manipulation method (%)** |  |  |  |  |  |  |  |
| Manipulated/Unmanipulated | 97/3% | 96/4% | 1.000 |  | 0/100% | 0/100% | N/A |
| **HCT-CI* (median [range])** | 0 (0, 1) | 0 (0, 2) | 0.277 |  | 0 (0, 1) | 0 (0, 1) | 0.435 |
| **Anthropometrical variables (mean ± SD)** |  |  |  |  |  |  |  |
| Body weight (kg) | 38.9 ± 25.1 | 40.3 ± 14.6 | 0.747 |  | 34.0 ± 15.7 | 36.4 ± 16.1 | 0.502 |
| BMI (kg/m^2^) | 19.7 ± 5.8 | 18.6 ± 3.6 | 0.319 |  | 17.6 ± 3.6 | 18.2 ± 3.2 | 0.654 |
| **Karnofsky/Lansky’s performance scale**** | 93 ± 7 | 94 ± 8 | 0.740 |  | 94 ± 5 | 92 ± 8 | 0.189 |

Significant differences (*p <* 0.05) between groups are in bold. Abbreviations: ALL, acute lymphoblastic leukemia; allo-HSCT, allogeneic hematopoietic stem cell transplantation; auto-HSCT, autologous hematopoietic stem cell transplantation; BMI, body mass index; CR, complete remission; GvHD, graft-versus-host disease; HCT-CI, hematopoietic cell transplantation-comorbidity index; HLA, human leukocyte antigens; HSCT, hematopoietic stem cell transplantation; N/A, not available; SD, standard deviation. Symbol: * comorbidities were calculated using the HCT-CI [10]; ** the Karnofsky and Lansky Play-Performance Scale for Pediatric Functional Status were used to assess the performance of children aged ≥ 16 or < 16 years, respectively, on a 0 to 100 ( "perfect") scale.
